# Supplementary material for: Healthcare professionals’ views on the most important outcomes for non-infectious uveitis of the posterior segment: A qualitative study
Source: PLoS One. 2023 Nov 17;18(11):e0294117. doi: 10.1371/journal.pone.0294117 (PMC10655978; doi:10.1371/journal.pone.0294117)
Supplement: S1 Table — (DOCX) [file pone.0294117.s001.docx]

Supplementary table 1: Questionnaire (Interviews)

## Defining a Core Outcome Set in patients with Uveitis both with and without Uveitic Macular Oedema

1. What is your job title / role?

Ophthalmologist  Policy Maker/Commissioner  Nurse practitioner Other (Specify)_________

1. Are you currently

Employed    Self-employed  Retired

1. Where is your Country of work?

______________________ (please specify)

1. How many years of experience do you have in the area of ophthalmology?

______________________

1. How many years of experience do you have in uveitis and UMO?

_________________

1. Have you contributed to clinical trials in uveitis?

Yes; if yes, please specify how many __________________________________  No

1. Do you have any experience in ophthalmology commissioning?

Yes; if yes, please specify your involvement_____________________________

No

1. Have you been involved with uveitis patient groups?

Yes (if yes please specify which group(s)_____________PINGU________________

No

**THANK YOU FOR TAKING THE TIME TO COMPLETE THIS QUESTIONNAIRE**
