# Supplementary material for: Healthcare professionals’ views on the most important outcomes for non-infectious uveitis of the posterior segment: A qualitative study
Source: PLoS One. 2023 Nov 17;18(11):e0294117. doi: 10.1371/journal.pone.0294117 (PMC10655978; doi:10.1371/journal.pone.0294117)
Supplement: Attachment — Submitted filename: Reviewers responses 211022.docx [file pone.0294117.s002.docx]

| **NO** | **COMMENT** | **AUTHORS’ RESPONSES** | **Line number in the manuscript** |
| --- | --- | --- | --- |
| **Reviewer 1** | | |  |
| **1** | Pg 6 – “As part of the study, “Development of a Core Outcome Set for Clinical Trials in Non-Infectious Uveitis affecting the Posterior Segment of the eye”, please cite reference | Thank you. Citation has now been inserted  As part of the study, “Development of a Core Outcome Set for Clinical Trials in Non-Infectious Uveitis affecting the Posterior Segment of the eye [1]. | 123-124 |
| **2** | The description of domains can be shortened down | We truly appreciate the reviewer suggestion, however for consistency and comprehensive presentation we kept the domain in that order which is the same order used in previous publications [1, 2]. Furthermore, we added a note previously to the methods about the relationship of this coding frame to the one presented in the previous paper to try to ensure this was clear. |  |
| **3** | How did the authors collect the patient and carer’s perspective on outcome. This should be clarified and added in methods | Thank you. In the data collection section we have already written text highlighted in yellow and we have made additional clarifications (highlighted pink):  *The collection of patients’ and carers’ perspectives were fully described in the reference [2]. (Tallouzi, M.O., Moore, D.J., Bucknall, N., Murray, P.I., Calvert, M.J., Denniston, A.K. and Mathers, J.M., 2020. Outcomes important to patients with non-infectious posterior segment-involving uveitis: a qualitative study. BMJ open ophthalmology, 5(1), p.e000481*).  A coding framework of outcome domains and items had already been developed during the analysis of previous focus group discussions with patients with NIU-PS and their carers [2]. The focus group data were coded inductively (from the raw data), without application of any *a priori* outcome domain framework and then applied to the interview data where relevant. New codes were devised where healthcare professionals discussed outcomes and related concepts that had not been identified previously by patients and carers. This coding framework was developed by two researchers (MOT and JMM). A sample of interviews were coded independently by MOT and JMM and the results of this were discussed in order to refine the coding framework. The final analysis was discussed amongst the broader research team.  A comparison of the outcome domains was undertaken using items identified from the interviews with healthcare professionals with those previously identified via focus groups with patients and carers (8) and from a systematic review of the effectiveness of the pharmacological agents for macular oedema associated with non-infectious uveitis of the posterior segment [3]. | 191-194 |
| **Reviewer 2** | | | |
| **1** | There are no line numbers in the manuscript which makes it hard to point out where corrections can be made | Thank you. Line numbers have been added |  |
| **2** | Introduction  Non infectious ......threatening forms of uveitis. Citation is needed | Thank you. Citations has been added  Non-infectious intermediate, posterior and panuveitis are the most sight-threatening forms of uveitis. These forms involve the posterior segment of the eye and are often grouped together in clinical trials, named as Non-Infectious Uveitis affecting the Posterior Segment (NIU-PS) [4, 5]. | 97 |
| **3** | What are examples of causes of NIU-PS. None are listed in the manuscript. | Thank you. We have inserted the following text  *Non-infectious uveitis which may be purely ocular or associated with a systemic disease (e.g. ankylosing spondylitis, Behcet’s disease or sarcoidosis [6, 7].* | 97-98 |
| **4** | Page 5..professional and compare these views with those.. | Thank you. This has been amended to read  It is therefore necessary to understand which outcomes matter to healthcare professionals’ *and compare their views* with those of patients and carers. | 115-116 |
| **5** | Page 6..agreed upon and are... | Thank you. The word upon has been added to the text  COSs are standardised sets of outcomes that have been scientifically *agreed upon* that are measured and reported in all trials for a specific clinical area that would promote consistency in outcome assessment [8]. | 116 |
| **6** | Page6-how valid is a phone interview compared to other methodologies? Why was this chosen? | Thank you. The following text has been added.  *Telephone interviews are introduced as a qualitative research method allowing researchers to have access to a wide geographical area and collect rich and in-depth data with evidence that they can produce equivalent data to face-to-face interviews” [9, 10].* | 177-179 |
| **7** | Healthcare professionals. Were other professionals considered? Optometrists? | Thank you. Response noted.  Other professionals were considered, but priority was given to those who were most involved in patient care and treatment decisions. Although first presentation of patients with PSIU may be to optometrists, almost all ongoing care (other than refraction) is with the specialist eye services within a hospital ophthalmic department (comprising ophthalmologists and nurse practitioners), and the availability of those treatments is dependent on the wider health system (commissioners). |  |
| **8** | Was the person who conducted the interviews trained appropriately on how to conduct the interview without bias and influence | Thank you. Response noted  The interview was conducted by the clinical research Fellow who underwent special training in qualitative methods including interviews and focus groups discussion and had been supervised by qualitative research expert. |  |
| **9** | You list 3 topic areas but maybe it should be 4? 3-patient expectations, 4 differences between patients and clinicians expectations | Thank you. This had been edited in the manuscript  *Four topics* were discussed in the interviews to understand healthcare professionals’ perspectives regarding the most important outcomes that should be assessed in clinical trials focusing on NIU-PS. These topic areas highlighted healthcare professionals’ thoughts on: i) the impact of uveitis on patients’ lives; ii) what outcomes are clinicians looking to achieve from the treatments provided for NIU-PS; iii) patients’ expectations from treatment; IV) whether there were differences between patients’ and clinicians’ expectations. Prior to the start of interviews, all participants were asked to complete a short background questionnaire that gathered data on sample characteristics and helped to promote diversity amongst the interview sample | 183 |
| **10** | a priori- I would try to avoid the use of Latin phrases | Thank you. the text has been amended to read  *A coding* framework of outcome domains and items had already been developed during the analysis of previous focus group discussions with patients with NIU-PS and their carers [2]. The focus group data were coded inductively (from the raw data), without application of any previous (preliminarily) outcome domain framework and then applied to the interview data where relevant | 202 |
| **11** | Page 9;15 participants-could you have recruited more? Is 15 considered significant? | Thank you. The following text has been added  *The sampling for this qualitative study was informed by a judgement of data saturation i.e. where the collection of new data no longer contributed additional elements to the outcome framework. Our conceptualisation of saturation was informed by the work of Cicely Kerr and colleagues specific to patient-reported outcome research [11].* | 162-165 |
| **12** | How much experience does one need to be considered an expert? Is 14 years enough? | Thank you. Response noted.  Expert in the field means a person with a broad and deep understanding and competence in terms of knowledge, skill and experience through practice and education in the field rather than how many years have done. | **Could you please check this** |
| **13** | Pages 10-33: some of the quotes are likely direct quotes but I would try to clean them up in terms of grammar. I'm not sure you need quotes for every single outcome in the manuscript itself. Perhaps it can be included as an appendix as it makes the manuscript very lengthy as written. | Thank you. Response noted.  We would prefer to present quotes verbatim as is the convention with most qualitative data, but would be happy to take editorial advice on this. |  |
| **14** | End of page 33-34: this paragraph needs to be re-written. Hard to read currently. | Response noted. Following the reviewer suggestion, this was rephrased.  *The disease control domain and its associated outcomes were discussed in all interviews. Clinicians’ highlight how important to assess the severity of the disease measuring its activity and link those findings to visual function. Healthcare professionals noted that treatment decisions were multi-factorial and included a consideration of the clinical findings including inflammatory markers, structural changes e.g. UMO and visual function and the patient’s associated functional ability. Considering all those components would allow a comprehensive assessment of patients’ health condition and provide a meaningful dialogue with patients, carers, and other stakeholders involved in the management of NIU-PS. It is crucial to understand whether an improvement in disease control outcomes would result in an associated positive impact on patients’ lives through improved functionality. For example, a patient is likely to notice a benefit after improved disease control if they have reversible macular oedema but not if that inflammation has become associated with a macular scar* | 686-696 |
| **Reviewer 3** | | | |
| **1** | The authors have evaluated the outcome measures for NIU-PS among healthcare professionals and patients/carers. The study methods and results are clear and well explained. Despite some limitations on the sample size and study population, as mentioned in the limitations, the submitted manuscript has great strengths. Some minor revisions are suggested for the manuscript which will bring further clarity.  1. The aim of the study is clear on the abstract. The abstract clearly mentions the methods and the findings of the study. However, for further clarity, it is advised to highlight the particular outcomes that the healthcare professionals placed more emphasis on. | Thank you. The following text has been added.  *Healthcare professionals recognise all of the outcome domains as patients/carers but with subtly differing emphasis within some domains and with a priority for certain types of measures. Healthcare professionals discussed the disease control and side effects/complications to a greater degree than patients and carers in the focus groups.* | 70-73 |
| **2** | Add references for the NIU-PS in the introduction. | Thank you. Citation has been added.  Non-infectious intermediate, posterior and panuveitis are the most sight-threatening forms of uveitis. These forms involve the posterior segment of the eye and are often grouped together in clinical trials, named as Non-Infectious Uveitis affecting the Posterior Segment (NIU-PS) [4, 5] | 97 |
| **3** | Delphi exercise is not defined or explained. Please add description with appropriate references. | Thank you for your suggestion. W have added further detail on the Delphi methodology to read as follow  As part of the study, “Development of a Core Outcome Set for Clinical Trials in Non-Infectious Uveitis affecting the Posterior Segment of the eye”, qualitative interviews were conducted to (a) understand the perspectives of healthcare professionals on what sort of outcomes they thought to be important for adult patients with non-infectious NIU-PS; and (b) contribute to the long-list of outcomes to feed into the Delphi exercise *(an online survey used to seek participants’ opinions were sought through two sequential rounds, with feedback from round 1 being provided. Participants would be asked to prioritise each outcome for inclusion in the study based on their level of importance using a nine-point Likert scale from 1 (no importance) to 9 (critically important));* (c) help the development of a COS for use in efficacy or effectiveness trials in adult patients with NIU-PS. The views of healthcare professionals were combined with those of patients and carers. Here we report the perspectives of healthcare professionals and compare these with the recently reported views of patients and carers with NIU-PS [2] | 127-130 |
| **4** | Research question is clearly outlined and the purpose of the study is justified with relevant background information. However, additional clarity is needed on what further will be achieved immediately and in the long run by understanding which outcomes matter to healthcare professionals vs patients/carers. If the authors make the long term directions of the study clearer and highlight how the results will be applicable, it will be helpful | Thank you. Additional text has been added  *Appreciating the different emphasis of healthcare professionals and patients will allow different stakeholder groups to appreciate where perspectives might differ, thereby encouraging inclusion of diverse opinions in core outcome set and other outcome-focused activities* | 118-120 |
| **5** | Some of the references are really old eg. Outcome measures on arthritis, chronic care models. Are there more recent studies for these topics? Please update with those. | Thank you. A new reference has been used  Kan, K., et al., Patients’ and clinicians’ perspectives on relevant treatment outcomes in depression: qualitative study. BJPsych open, 2020. **6**(3). | 123 |
| 1. Tallouzi, M.O., et al., *Healthcare Professionals’ Views on the Most Important Outcomes for Non-Infectious Uveitis of the Posterior Segment: A Qualitative Study.* 2021.  2. Tallouzi, M.O., et al., *Outcomes important to patients with non-infectious posterior segment-involving uveitis: a qualitative study.* BMJ open ophthalmology, 2020. **5**(1): p. e000481.  3. Tallouzi, M.O., et al., *The effectiveness of pharmacological agents for the treatment of uveitic macular edema (UMO): A systematic review.* Ocular immunology and inflammation, 2019. **27**(4): p. 658-680.  4. Mikhail, M. and A. Sallam, *Novel intraocular therapy in non-infectious uveitis of the posterior segment of the eye.* Medical Hypothesis, Discovery and Innovation in Ophthalmology, 2013. **2**(4): p. 113.  5. Merrill, P.T., et al., *Efficacy and Safety of Intravitreal Sirolimus for Non-Infectious Uveitis of the Posterior Segment: Results from the SAKURA Program.* Ophthalmology, 2020.  6. Jones, N. and N.P. Jones, *Uveitis*. 2012: JP Medical Ltd.  7. Barry, R.J., et al., *Pharmacotherapy for uveitis: current management and emerging therapy.* Clinical ophthalmology (Auckland, NZ), 2014. **8**: p. 1891.  8. COMET Initiative. *Core Outcome Measures in Effectiveness Trials 2011. Available from:* [*http://www.comet-initiative.org/*](http://www.comet-initiative.org/).  9. Ritchie, J., et al., *Qualitative research practice: A guide for social science students and researchers*. 2013: sage.  10. Novick, G., *Is there a bias against telephone interviews in qualitative research?* Research in nursing & health, 2008. **31**(4): p. 391-398.  11. Kerr, C., A. Nixon, and D. Wild, *Assessing and demonstrating data saturation in qualitative inquiry supporting patient-reported outcomes research.* Expert review of pharmacoeconomics & outcomes research, 2010. **10**(3): p. 269-281. | | | |
|  | | | |

**Editor’s comments**

Please ensure that your manuscript meets PLOS ONE's style requirements, including those for file naming. The PLOS ONE style templates can be found at

https://journals.plos.org/plosone/s/file?id=wjVg/PLOSOne_formatting_sample_main_body.pdf and

https://journals.plos.org/plosone/s/file?id=ba62/PLOSOne_formatting_sample_title_authors_affiliations.pdf

2. We note that the grant information you provided in the ‘Funding Information’ and ‘Financial Disclosure’ sections do not match.

When you resubmit, please ensure that you provide the correct grant numbers for the awards you received for your study in the ‘Funding Information’ section.

**Funding**

This article represents an independent research project funded by the National Institute for Health Research (NIHR) under the Program Clinical Doctoral Research Fellowship Scheme at the University of Birmingham Award reference: CDRF-2014-05-057 ([www.nihr.ac.uk](http://www.nihr.ac.uk)). The views and opinions expressed by authors in this publication are those of the authors and not necessarily those of the NHS, the NIHR or the Department of Health. The funders had no role in study design, data collection and analysis, decision to publish, or preparation of the manuscript.

**Competing Interests**

On behalf of all authors, I disclose that all named authors declare that they have no competing interests exist

3. Thank you for stating the following in the Acknowledgments/ Funding Section of your manuscript:

This article represents an independent research project funded by the National Institute for Health Research (NIHR) under the Clinical Doctoral Research Fellowship Program. The views and opinions expressed in this publication are those of the authors and not necessarily those of the NHS, the NIHR or the Department of Health and Social Care.

We note that you have provided additional information within the Acknowledgements Section that is not currently declared in your Funding Statement. Please note that funding information should not appear in the Acknowledgments section or other areas of your manuscript. We will only publish funding information present in the Funding Statement section of the online submission form.

Please remove any funding-related text from the manuscript and let us know how you would like to update your Funding Statement. Currently, your Funding Statement reads as follows:

The funders had no role in study design, data collection and analysis, decision to publish, or preparation of the manuscript.

Please include your amended statements within your cover letter; we will change the online submission form on your behalf.

4. Please note that in order to use the direct billing option the corresponding author must be affiliated with the chosen institute. Please either amend your manuscript to change the affiliation or corresponding author, or email us at plosone@plos.org with a request to remove this option.

5. Your ethics statement should only appear in the Methods section of your manuscript. If your ethics statement is written in any section besides the Methods, please delete it from any other section.

Format for specific study types Human Subject Research (involving human participants and/or tissue) Give the name of the institutional review board or ethics committee that approved the study • Include the approval number and/or a statement indicating approval of this research • Indicate the form of consent obtained (written/oral) or the reason that consent was not obtained (e.g. the data were analyzed anonymous

**Ethical approval**

Ethical approval for the study has been granted by the National Research Ethics Service (NRES) West Midlands –South Birmingham Research Ethics Committee (Reference number 17-WM-0111). Verbal recorded consent has been obtained

**Data availability**

All relevant data are within the manuscript and its Supporting Information files
