# Supplementary material for: Healthcare professionals’ views on the most important outcomes for non-infectious uveitis of the posterior segment: A qualitative study
Source: PLoS One. 2023 Nov 17;18(11):e0294117. doi: 10.1371/journal.pone.0294117 (PMC10655978; doi:10.1371/journal.pone.0294117)
Supplement: Attachment — Submitted filename: Reviewers responses October 23.docx [file pone.0294117.s004.docx]

Institute of Clinical Sciences

University of Birmingham

Edgbaston

Birmingham

B15 2TT

3^rd^ October 2023

Dear Diviacchi

**Healthcare Professionals’ Views on the Most Important Outcomes for Non-Infectious Uveitis of the Posterior Segment: A qualitative study**

The authors would like to thank the reviewers and editor for their constructive comments and the opportunity to improve the manuscript.

As previously stated we have revised the reviewers’ comments, and we can confirm that all comments have answered in the previous submitted version as outlined below. All edits have been highlighted in track changes in the main manuscript. We believe that the manuscript is strengthened as a result and hope that it is now suitable for publication in Plos One.

I have included all reviewers’ comments and authors’ responses. We are aware that no further comments made by reviewer 1 & 2 in the second round. However, reviewer 3highlited that we have partially addressed his comments and no responses regarding the principal component analysis (PCA), data analysis, the coding framework. Please refer to the authors responses below and the added detailed to the manuscript

We look forward to hearing from you,

Dr Mohammad Tallouzi

| **NO** | **COMMENT** | **AUTHORS’ RESPONSES** | | **Line number in the manuscript** |
| --- | --- | --- | --- | --- |
| **Reviewer 1** | | | |  |
| **1** | There are some grammatical or word choices within the manuscript, particularly in the introduction, which impact readability overall, with some examples excerpted in the specific comments.  Line 97-98: The highlighted sentence: Delete “which”  Line 106-108: The use of the word “whilst” at the beginning of this sentence suggests that a comparison is going to occur in the sentence which does not occur, rendering the sentence somewhat confusing. Similar grammatical errors in the introduction are seen repeatedly, impacting readability | | Thank you, these have been corrected and highlighted in track changes.  Thank you - deleted  Thank you- deleted. | 97  106 |
| **2** | Study Design: please expand on this in this area beyond simply stating that this was “a qualitative research study” | | Thank you. We have inserted the following text:  This was a qualitative research study using semi-structured telephone interviews. It was a component of a broader development of a core outcome set (COS) in noninfectious PSIU (COSUMO) (17, 20,21). | 139-140 |
|  | Line 176: Telephone interviews are introduced as a qualitative research method allowing researchers to have access to a wide geographical area and collect rich and in-depth data with evidence that they can produce equivalent data to face-to-face interviews” Is this sentence a quote or not? There are quotation marks at the end of this highlighted sentence so it is not clear if these are the words of the authors or the reference being cited | | Thank you for pointing this out and you are correct that this is not a quote. The quotation marks have now been removed | 188 |
|  | Line 227-228: Please explain what the “(8)” signifies at the end of this sentence: A total of 43 separate outcomes were interpreted and mapped onto the 11 previously reported outcome domains (8) | | Thank you. This was a typographical error and it meant to refer to the reference #11 which is the published paper Tallouzi, M.O., et al., Outcomes important to patients with non-infectious posterior segment-involving uveitis: a qualitative study. BMJ open ophthalmology, 2020. 5(1): p. e000481.  This has been amended. | 250 |
| **Reviewer 2** | | | | |
| **1** | Abstract line 70 “Healthcare professionals recognize all of the outcome domains as patients/carers….”  Please modify to indicate the patient/carers outcome domains were from a different and published study | Thank you. This has been amended and now read  Healthcare professionals recognize all of the published outcome domains as patients/carers in the previous publication (11) but with subtly differing emphasis within some domains and with a priority for certain types of measures. Healthcare professionals discussed the disease control and side effects/complications to a greater degree than patients and carers in the focus groups. | | 70-73 |
| **2** | The Introduction provides the rationale for the study as expected, although the final section does not clearly state that the patient and carer responses were published previously – please include this reference and modify sentence as below. Thank you.  Line 131 “The views of healthcare professionals were combined with those of patients and carers [2], and we now report and compare the perspectives of healthcare professionals the recently reported views of patients and carers with NIU-PS [2].” | Thank you. This has been amended according to the reviewer’s comments and now reads:  The views of healthcare professionals were combined with those of patients and carers (11), and we now report and compare the perspectives of healthcare professionals to the recently reported views of patients and carers with PSIU (11). | | 133-136 |
| **3** | The details of the practitioner participants are provided in Table 1, but exclusion criteria should be noted. The reasons for not including other eye care practitioners such as optometrists may also be useful to comment on in the manuscript | Thank you. Patients with their first attack of uveitis (of any type) may initially seek help from a general practitioner or an optometrist but more frequently attend (self-refer) an Eye A&E department. General practitioners have limited ophthalmological expertise and the appropriate equipment to diagnose or manage patients with uveitis. They may immediately refer the patient to an optometrist but more likely to an Eye A&E department. Optometrists may see patients with PSIU but this would be at their first presentation and then are required to refer the patient to an Eye A&E department (<https://www.college-optometrists.org/clinical-guidance/clinical-management-guidelines/uveitis_anterior>). Once a patient is under the Hospital Eye Service then all further ophthalmological is undertaken by the hospital and not in the primary care setting. If patients have recurrences or flare-ups of their uveitis, they will contact the hospital they are under to be seen sooner than arranged. The following text has now been added to the manuscript:  Exclusion criteria  General practitioners and optometrists were excluded from the study. General practitioners have limited ophthalmological expertise and also lack the appropriate equipment to diagnose or manage patients with uveitis. Although the first presentation of patients with PSIU may be to optometrists, almost all ongoing care (other than refraction) is with the specialist eye services within a hospital ophthalmic department (comprising ophthalmologists and nurse practitioners), and the availability of those treatments is dependent on the wider health system (commissioners). | | 148-153 |
| **4** | Were the practitioners all urban-based? An explanation of commissioners would also be helpful as this role is not available in all countries. | Thank you.  All practitioners were urban-based consultant ophthalmologists with a special interest in uveitis tend to work in large eye units in major cities and patients are seen there rather than at smaller, peripheral, rural eye units.  Participants were deemed eligible for telephone interviews if they were healthcare professionals (e.g. ophthalmologists, nurse practitioners) involved in caring for patients with PSIU, or health policy-makers/commissioners who were involved in commissioning with an influence on uveitis care or policy-making in the area of ophthalmology | | 146-148 |
| **5** | How many practitioners, commissioners and nurses were contacted for the final number of 12 participants? | Thank you. Further details were added to read  Initially the doctoral research fellow recruited 7 ophthalmologists, 5 nurse practitioners and 6 health policy-makers/commissioners , however, the telephone interviews were completed with 5 ophthalmologists, 3 nurse practitioners and 4 health policy-makers/commissioners. | | 240-243 |
| **6** | The authors also note on line 187: | Thank you. A colon has now been deleted. | | 196 |
| **7** | “Prior to the start of interviews, all participants were asked to complete a short background questionnaire that gathered data on sample characteristics and helped to promote diversity amongst the interview sample.” Please provide details of this additional survey as a supplementary file – what characteristics were surveyed? | Thank you. Details of the survey have been added to the manuscript as supplementary file | | 696-728 |
| **8** | Were the differences in participant characteristics such as years of experience and clinical trials experience, for example, considered when analysing the findings from the study? How was experience 'measured'? | Thank you.  Experience was measured by the number of years specialising in the field of uveitis. We did not notice any clear pattern in the data according to clinical or clinical trials experience. The majority of participants articulated the broad range of outcomes outlined in this paper | |  |
| **9** | The interviews were conducted in 2018 and wonder if there are concerns as to how the comments from then, may relate to the most recent patterns of clinical care for NIU-PS patients, including what has happened with these patients during COVID? Can the authors please comment and include mention in limitations related to this aspect of their research | Thank you. This is an important point. Although there was considerable disruption in hospital services during the peaks of COVID-19, ophthalmologists continued to see patients face-to-face (albeit in reduced numbers) unlike many other specialties that only undertook telephone or video consultations. The true impact of the COVID-19 pandemic and vaccination on patients with PSIU is outside the scope of this paper but would be an interesting piece of research for the future.  We have now included a sentence in the Discussion stating this as a potential limitation:  Although the pandemic has caused disruption to clinical care patterns that we would anticipate those outcomes that matter to healthcare professionals may remain largely unchanged, however further research would be required to confirm this | | 795-799 |
| **10** | For the 11 outcome domains included, the authors indicate these were the same domains as those for the patients and carers. In Table 3, these outcome domains and items are then compared for practitioners and patients and carers. Please reference the published study in the column for patients and carers (ref 11). A systematic review is also referred to in Table 3, and this should also be referenced. | Thank you. Citations have now been inserted in table 3 and the headings for columns 3 and 4 now read  Patients/carers (11)  Systematic review (10) | | 676 |
| **11** | References require attention to detail. Please see examples below (and there may be more). Please check these carefully. | Thank you. The references have been updated | |  |
| **Reviewer 3** | | | | |
| **1** | In this study the Tallouzi and colleagues canvassed a number of clinicians on their views on what they thought were the most important outcomes in the management of non-infectious uveitis, then compared their results with systematic reviews and their past work exploring a similar field with patient and carer groups.  Whist it is clear that an extensive amount of work has been done, with a lot of data collected, it is unclear how their data was actually analysed, largely due to how it has been presented to the reader. | | Thank you. This is described in the data analysis section and below:  This is a qualitative research study by which data were analysed using thematic analysis informed by the framework analytical approach (23,25). Interviews were digitally audio-recorded and transcribed clean verbatim by an external specialist transcription service. The clinical doctoral research fellow (MOT) checked the quality of the transcription and corrected any mistakes against the recordings by listening to each audio recording and reading the transcript repeatedly to become familiar with the data. A coding framework of outcome domains and items had already been developed during the analysis of previous focus group discussions with patients with PSIU and their carers (11). The focus group data were coded inductively (from the raw data), without application of any *a priori* outcome domain framework and then applied to the interview data where relevant. New codes were devised where healthcare professionals discussed outcomes and related concepts that had not been identified previously by patients and carers. This coding framework was developed by two researchers (MOT and JMM). A sample of interviews was coded independently by MOT and JMM and the results of this were discussed in order to refine the coding framework. The final analysis was discussed amongst the broader research team.  A comparison of the outcome domains was undertaken using items identified from the interviews with healthcare professionals with those previously identified via focus groups with patients and carers (11) and from a systematic review of the effectiveness of the pharmacological agents for macular oedema associated with non-infectious uveitis of the posterior segment (2) | 208-236 |
| **2** | In the methods, the authors describe a framework analytical approach that they used in their similar prior work. Although such a framework has been published previously, a brief description in the methodology of this paper should also be provided. | | Thank you.  The framework has been used in our previous qualitative research work informed by data collected from the focus group discussions describing outcomes important to patients with Non-infectious Posterior Segment-Involving Uveitis (11).  Please refer to our response (1) above regarding further analytical detail. | 209-211 |
| **3** | The authors also describe a coding framework of outcome domains, but do not enlighten the reader as to how this coding was done, and thus how the data was actually analysed | | Thank you. Further detail on the analysis has been provided:  Interviews were digitally audio-recorded and transcribed clean verbatim by an external specialist transcription company, and subsequently checked for quality and anonymised by the interviewer. Data were analysed using an inductive thematic approach, following the six steps proposed by Braun and Clarke.  Initially, transcripts were read repeatedly to aid familiarisation and allow for data immersion. This facilitated the generation of preliminary codes and themes supported by the use of NVivo software, which eventually progressed into a developed coding frame. Two authors coded percentage of the transcripts independently to cross-check the coding strategy. During this process our definition of an outcome was broad, including any consequence of PSIU or its treatment that clearly had significance to patients and carers who participated in the focus groups. Once we had finalised our coding framework it was then applied to the whole dataset (indexing). Data were then summarised descriptively retaining the original meaning of participants’ discussions. Two of the most concept-rich transcripts were independently double-coded by two authors (MOT and JMM) and additional interpretations were incorporated into the coding frame. These early analytic findings were then discussed amongst the research team and agreement of the final themes was gained | 211-226 |
| **4** | Whilst a comparison of outcome domains with their prior work with patient and carer focus groups is described by the authors, this appears to be a tick box exercise, rather than a rigorous scoring of congruity (or otherwise) within each domain. . | | Thank you. Response noted  Table 2 described in details outcome domains as discussed by healthcare professionals, however table 3 provides a comparison of the outcome domains and items identified from the interviews with healthcare professionals with those previously identified via focus groups with patients and carers (11) and a systematic review of the effectiveness of the pharmacological agents for macular oedema associated with non-infectious uveitis of the posterior segment (10).  Table 3 highlights the fact that healthcare professionals discussed all of the outcome domains that had previously been identified via focus group discussions with patients and carers, but with some differences in items within certain domains. However, only visual function (distance vision), treatment side effects and disease control outcomes were assessed in previous clinical research identified as part of the systematic review | 257-262  654-658 |
| **5** | One therefore wonders whether the authors have considered analysing their data using a principal component analysis (PCA) to assess the construct validity of their survey questions and determine whether the outcomes were consistent with the desired constructs. The advantage of a PCA is that it is a proven method to simplify the dimensionality of multivariate qualitative data into a limited number of new variables, allowing such multivariate data to be better visualised graphically & therefore easier to analyse overall  This may therefore reduce the large number of domains presented by the authors in their results, as currently, as presented, what the authors are essentially presenting are a whole lot of quotes grouped into 11 different categories, which is essentially quite hard to summarise into a clear message, particularly when each field is then further subdivided (as per Table 3).  Given the large amount of collected data, the lack of a clear message is a shame, hence the suggestion to consider alternate ways of analysing and thus presenting, these results.  Has the statistical analysis been performed appropriately and rigorously? | | Thank you  We think that the reviewer may have misunderstood the method slightly (due to our lack of detail, which has now been provided). These qualitative research methods are a recognised part of COS development (17). These types of qualitative research approaches produce in-depth spoken data suitable for the type of qualitative research analysis described (12,15). We did not start out with survey questions or desired constructs. Rather qualitative methods use participant-focused and very open ended discussion techniques to elucidate participants’ thoughts and perspectives. The spoken data produced are then analysed using qualitative research analysis such as those also used in the conceptual elicitation in the development of patient-reported outcomes. These analytical techniques are well recognised and recommended for use with these types of data (18). The desire is not to simplify and reduce these data, but rather to represent the data in it’s underlying complexity. Hence the categories with sub-components. This is very different to analysis of datasets and objectives of that analysis that for example PCA would be applicable to.  This is qualitative data and statistical analysis is not applied as stated above |  |

| **NO** | **COMMENT** | **Reviewers comments** | **AUTHORS’ RESPONSES** |
| --- | --- | --- | --- |
| **1** | If the authors have adequately addressed your comments raised in a previous round of review and you feel that this manuscript is now acceptable for publication, you may indicate that here to bypass the “Comments to the Author” section, enter your conflict of interest statement in the “Confidential to Editor” section, and submit your "Accept" recommendation. | Reviewer #1: All comments have been addressed  Reviewer #3: (No Response) | Thanks you  All comments have been addressed |
| **2** | Is the manuscript technically sound, and do the data support the conclusions? | Reviewer #1: Yes  Reviewer #3: No | Thank you  No further details given by reviewer 3 why the data do not support the conclusion |
| **3** | Has the statistical analysis been performed appropriately and rigorously | Reviewer #1: N/A  Reviewer #3: No | Thank you  As stated above statistical analysis do not apply to qualitative data, however, these types of qualitative research approaches produce in-depth spoken data suitable for the type of qualitative research analysis described |
|  | Have the authors made all data underlying the findings in their manuscript fully available? | Reviewer #1: Yes  Reviewer #3: Yes | Thank you |
| **4** | 5. Is the manuscript presented in an intelligible fashion and written in standard English? | Reviewer #1: Yes  Reviewer #3: Yes | Thank you |
| **5** | Is the manuscript presented in an intelligible fashion and written in standard English? | Reviewer #1: Yes  Reviewer #3: Yes | Thank you |
| **6** | Review Comments to the Author  Please use the space provided to explain your answers to the questions above. You may also include additional comments for the author, including concerns about dual publication, research ethics, or publication ethics. (Please upload your review as an attachment if it exceeds 20,000 characters) | Reviewer #1: (No Response)  Reviewer #3: As presented, the authors have addressed some of the reviewer comments, but have chosen to ignore others. Part of the reason according to the author response is that the authors wish to present their findings in the same way as their prior publication. However, the suggestions put forward by the reviewers does not preclude this - they are suggestions that may further enhance the presentation of their results with greater statistical validity, but these have not been directly addressed in this submission | Thank you  I confirm that all comments have been answered in the table above  I have included all responses to all reviewers. I am aware that reviewer 2 has not responded in the second round and reviewer 1 has no further comments to add |

**Editor’s comments**

1) **The use of non standard acronyms should be avoided (e.g., NIU-PS); I note that a different acronym (PSIU) was used in the complementary article published in 2022 in BMJ Open Ophthalmology. To avoid confusion "non-infectious uveitis of the posterior segment" should not be shortened.**

Although we used NIU-PS for the Ophthalmology paper, I have changed NIU-PS to PSIU for consistency with the other paper (2020 ) plus it is mentioned so many times in the manuscript that it would be rather unwieldy to use "non-infectious uveitis of the posterior segment" for each occurrence.

**2) We note that the grant information you provided in the ‘Funding Information’ and ‘Financial Disclosure’ sections do not match.**

**When you resubmit, please ensure that you provide the correct grant numbers for the awards you received for your study in the ‘Funding Information’ section.**

**Funding**

This article represents an independent research project funded by the National Institute for Health Research (NIHR) under the Program Clinical Doctoral Research Fellowship Scheme at the University of Birmingham Award reference: CDRF-2014-05-057 ([www.nihr.ac.uk](http://www.nihr.ac.uk)). The views and opinions expressed by authors in this publication are those of the authors and not necessarily those of the NHS, the NIHR or the Department of Health. The funders had no role in study design, data collection and analysis, decision to publish, or preparation of the manuscript.

**3. Your ethics statements should appear in the method section of your manuscript**

This has section has now been moved to the method section

**Ethical approval (**ethics statement should only appear in the Methods section of your manuscript)

Ethical approval for the study has been granted by the National Research Ethics Service (NRES) West Midlands –South Birmingham Research Ethics Committee (Reference number 17-WM-0111). Verbal recorded consent has been obtained
